# Evolving therapeutic landscape of diffuse large B-cell lymphoma: challenges and aspirations

**DOI:** 10.1007/s12672-023-00754-8

**Published:** 2023-07-19

**Authors:** Jason Yongsheng Chan, Nagavalli Somasundaram, Nicholas Grigoropoulos, Francesca Lim, Michelle Limei Poon, Anand Jeyasekharan, Kheng Wei Yeoh, Daryl Tan, Georg Lenz, Choon Kiat Ong, Soon Thye Lim

**Affiliations:** 1 Division of Medical Oncology, National Cancer Centre Singapore, 30 Hospital Blvd, Singapore, 168583 Singapore; 2 Cancer and Stem Cell Biology, Duke-NUS Medical School, National Cancer Centre Singapore, 8 College Road, Singapore, 169857 Singapore; 3 Department of Haematology, Singapore General Hospital, Singapore, Singapore; 4 Department of Haematology, National University Cancer Institute, Singapore, Singapore; 5 Cancer Science Institute of Singapore, National University of Singapore, Singapore, Singapore; 6 Department of Haematology-Oncology, National University Cancer Institute, Singapore, Singapore; 7 Division of Radiation Oncology, National University Cancer Institute, Singapore, Singapore; 8 Mount Elizabeth Novena Hospital, Singapore, Singapore; 9 Department of Medicine A, Department of Hematology, Oncology and Pneumology, University Hospital Münster, Münster, Germany; 10 Lymphoma Genomic Translational Research Laboratory, Cellular and Molecular Research, National Cancer Centre Singapore, Singapore, Singapore

**Keywords:** Lymphoma, Genomics, Antibody–drug conjugate, Precision oncology, CAR-T

## Abstract

Diffuse large B-cell lymphoma (DLBCL) represents the commonest subtype of non-Hodgkin lymphoma and encompasses a group of diverse disease entities, each harboring unique molecular and clinico-pathological features. The understanding of the molecular landscape of DLBCL has improved significantly over the past decade, highlighting unique genomic subtypes with implications on targeted therapy. At the same time, several new treatment modalities have been recently approved both in the frontline and relapsed settings, ending a dearth of negative clinical trials that plagued the past decade. Despite that, in the real-world setting, issues like drug accessibility, reimbursement policies, physician and patient preference, as well as questions regarding optimal sequencing of treatment options present difficulties and challenges in day-to-day oncology practice. Here, we review the recent advances in the therapeutic armamentarium of DLBCL and discuss implications on the practice landscape, with a particular emphasis on the context of the healthcare system in Singapore.

## Introduction

Diffuse large B-cell lymphoma (DLBCL) represents the commonest subtype of non-Hodgkin lymphoma and encompasses a group of diverse disease entities, each harboring unique molecular and clinico-pathological features. The recent fifth edition of the World Health Organization (WHO) Lymphoma Classification as well as the International Consensus Classification (ICC) recognizes 17 subcategories of large B-cell lymphomas other than DLBCL, not otherwise specified (DLBCL, NOS), reflecting their broad clinical spectrum and biological complexity [1, 2]. Nonetheless, the heterogeneity of DLBCL remains incompletely represented in the current framework, as the molecular genetics of the disease and their clinical actionability continue to be investigated and refined.

Contemporarily, approximately two-thirds of patients diagnosed with DLBCL can achieve durable remission and be cured after frontline treatment with rituximab-based chemoimmunotherapy. For the remaining patients however, autologous stem-cell transplantation (ASCT) [3, 4] may provide a chance of cure in only a quarter of the cases, and survival outcomes for early relapsed or refractory DLBCL remain dismal [5, 6]. Remarkably, in terms of treatment modalities, several newer options have been recently approved and are now available both in the first-line and relapsed settings, ending a dearth of negative clinical trials that plagued the past decade [7]. Despite that, in the real-world setting, issues like drug accessibility, reimbursement policies, physician and patient preference, as well as questions regarding optimal sequencing of treatment options present difficulties and challenges in day-to-day oncology practice.

Additionally, even as the therapeutic landscape continues to evolve rapidly with the anticipated entry of novel agents like bispecific T-cell engagers (BiTE), antibody–drug conjugates (ADC), multitarget-specific drugs, and innovative immunotherapies, a daunting task remains in the clinical integration of information derived from our improved understanding of the molecular pathology and genomic composition of DLBCL, with the ultimate goal of delivering precision medicine in the clinic [8, 9]. In this paper, we review the rapid advances in the therapeutic armamentarium of DLBCL and discuss implications on the practice landscape in the context of the evolving healthcare system in Singapore.

## DLBCL in the Singapore context

Singapore is a developed country in Asia with a population of over 5 million people, comprising multi-ethnic representatives from East (Chinese), South-East (Malay) and South Asian (Indian) ethnicities where each group has their unique sociocultural characteristics and genetic background. In keeping with global cancer trends, lymphomas are the fifth most common cancer in males and sixth most common cancer in females in Singapore [10, 11]. According to earlier statistics (from 1998 to 2007), the incidence of lymphoma in Singapore has been increasing at an average rate of 2.4% and 4.0% per year in males and females, respectively [12]. In a previously published population-based cancer registry study, DLBCL was the most common subtype amongst the mature B-cell lymphomas, accounting for about 30% of all lymphoid neoplasms from 1998 to 2012 [12]. In terms of first-line treatment, anti-CD20 monoclonal antibody rituximab plus cyclophosphamide, doxorubicin, vincristine, prednisolone (R-CHOP) chemoimmunotherapy has remained standard of care for the past two decades, consistent with international practice guidelines [13, 14].

Recently, the financing scheme of cancer treatment in Singapore had been refined in view of its rising costs and to ensure insurance premiums remain affordable over time. A Cancer Drug List (CDL) has been introduced by the Ministry of Health (MOH) of Singapore, incorporating the concept of limited co-payment together with comprehensive accessibility for approved cancer therapies used in Singapore. This includes most of the standard approved therapies for lymphoma (Table 1). For a specific drug to be on the list, pharmaceutical companies are responsible for demonstrating the clinical and cost-effectiveness of the treatment through the Agency for Care Effectiveness (ACE), Singapore’s national Health Technology Assessment (HTA) agency. Based on the evidence and companies’ price proposals, the MOH Drug Advisory Committee (DAC), chaired by the Director of Medical Services in MOH and comprising senior public sector doctors, pharmacists and MOH representatives, makes recommendations to MOH for listing and reimbursement limits. The MediShield Life scheme is a compulsory national basic health insurance scheme, while the MediSave scheme is a national medical savings scheme, both of which have predetermined claim and withdrawal limits, respectively. In public healthcare institutions, listed drugs may be subsidised in two frameworks—the Standard Drug List (SDL) and the Medication Assistance Fund (MAF), both largely determined by the patient’s monthly per capita household income. Cancer treatments that are not listed on the CDL are otherwise not readily eligible for reimbursement [15].

**Table 1:** Cancer Drug List (CDL) for lymphoma treatment in Singapore (as of 1 Apr 2023)

Drug name	Clinical indication
Acalabrutinib	Mantle cell lymphoma; received at least one prior therapy
Bexarotene	Cutaneous T-cell lymphoma; received at least one prior systemic therapy
Brentuximab vedotin	CD30 + peripheral T-cell lymphoma; previously untreated
Brentuximab vedotin	CD30 + cutaneous T-cell lymphoma; received at least 1 prior systemic therapy
Brentuximab vedotin	Relapsed or refractory CD30 + Hodgkin lymphoma; following ASCT or at least two prior therapies when ASCT or multi-agent chemotherapy is not an option
Brentuximab vedotin	Relapsed or refractory systemic anaplastic large cell lymphoma
Brentuximab vedotin	Consolidation treatment of CD30 + Hodgkin lymphoma who are at increased risk of relapse or progression following ASCT
Brentuximab vedotin	Previously untreated CD30 + advanced classic Hodgkin lymphoma
Copanlisib	Relapsed follicular lymphoma who have received at least two prior systemic therapies
Crisantaspase	Hypersensitivity to E.coli-derived asparaginase
Crizotinib	Relapsed or refractory, systemic ALK-positive anaplastic large cell lymphoma
Ibrutinib	Waldenstrom’s Macroglobulinaemia
Ibrutinib	Mantle cell lymphoma; received at least one prior therapy
Idelalisib	Relapsed follicular lymphoma; received at least two prior systemic therapies
Nivolumab	Relapsed or refractory classical Hodgkin lymphoma after ASCT and treatment with brentuximab vedotin
Obinutuzumab	Follicular lymphoma that has not responded to or progressed within 6 months after treatment with rituximab or a rituximab-containing regimen
Obinutuzumab	Previously untreated stage II bulky, III or IV follicular lymphoma
Pembrolizumab	Treatment of patients with relapsed or refractory classical Hodgkin lymphoma, who have failed ASCT or following at least two prior therapies when ASCT is not a treatment option
Pembrolizumab	Refractory primary mediastinal B-cell lymphoma, or who have relapsed after 2 or more prior lines of therapy
Rituximab (subcutaneous)	Maintenance treatment of follicular lymphoma who have responded to induction therapy
Rituximab (subcutaneous)	CD20 + diffuse large B-cell non-Hodgkin lymphoma
Rituximab (subcutaneous)	Previously untreated stage III-IV follicular lymphoma
Romidepsin	Cutaneous T-cell lymphoma; received at least one prior systemic therapy

*ASCT* autologous stem cell transplant

On March 18 2023, the Singapore Lymphoma Scientific Symposium 2023 was convened at the Academia, Singapore General Hospital campus, involving major stakeholders in lymphoma care across the country. A closed session was held by an invited panel of lymphoma experts across Singapore, in which the local management practices on DLBCL care in various settings were discussed and documented. Lymphoma specialists from both public (co-authors J.Y.C., N.S., N.G., F.L. M.L.P., A.J., K.W.Y., S.T.L.) and private (D.T.) practice were included, as well as the lead lymphoma scientist of the Singapore Lymphoma Study Group (C.K.O.). Viewpoints from an international expert outside of Singapore (G.L.) were also obtained and discussed with respect to the local context. These perspectives have been summarized and relevant aspects of local practice preferences for DLBCL are included in this article.

## Current first line treatment of DLBCL

### Establishment of R-CHOP as standard first-line therapy

Coiffier et al. first published the results of the seminal LNH-98.5 randomized trial in 2002, providing evidence for improved overall survival (OS) outcomes in 399 previously untreated elderly patients with DLBCL in the age range of 60–80 years. In this study, patients assigned to receive eight cycles of R-CHOP every 3 weeks had significantly improved complete response rates compared to those who received eight cycles of CHOP (76% vs 63%, respectively). Importantly, R-CHOP treatment resulted in better event-free survival (EFS) and OS outcomes without greater toxicity [16], leading to incorporation of rituximab into the standard first-line therapy for DLBCL. With a median follow-up of 10 years, the 10-year progression-free survival (PFS) was 36.5% with R-CHOP, compared with 20% with CHOP alone. The 10-year OS was 43.5% and 27.6%, respectively [17]. Pfreundschuh et al. reported the results of the MInT study in 2006, in which 824 young patients aged 18–60 years with DLBCL received six cycles of CHOP-like chemotherapy with or without rituximab. In keeping with findings from elderly patients, the addition of rituximab led to higher complete response rates, as well as increased EFS and OS [18]. With a median follow-up of 72 months, the 6-year EFS was 74.3% and 55·8%, while the 6-year OS was 90.1% and 80.0%, for patients treated with and without rituximab, respectively [19]. The RICOVER-60 study subsequently showed no difference in outcomes comparing six and eight cycles of R-CHOP given every 2 weeks [20]. These studies firmly established six cycles of R-CHOP as standard first-line therapy for DLBCL.

### Beyond standard R-CHOP therapy

Since the early pivotal trials that led to the adoption of anti-CD20 monoclonal antibody-based chemoimmunotherapy R-CHOP as standard first-line treatment of DLBCL [16–20], further progress in the field has been hampered by a succession of failed phase III clinical trials attempting to improve upon it. Dose-intensified induction regimens such as R-ACVBP (rituximab, doxorubicin, cyclophosphamide, vindesine, bleomycin, and prednisone), while providing significant EFS and OS benefit, is logistically complex and carries significant risk of haematological toxicities. The study population was also limited to young patients aged 18–59 years with age-adjusted international prognostic index (aa-IPI) scores of 1, precluding widespread clinical adoption [21]. Other intensification strategies including the use of dose-adjusted EPOCH-R (DA-EPOCH-R; etoposide, prednisone, vincristine, cyclophosphamide, doxorubicin, and rituximab) [22], as well as consolidation with high-dose chemotherapy (HDC) and ASCT [23–25] have not been demonstrated in a prospective randomized controlled trial to provide significant benefit over R-CHOP. In addition, the incorporation of novel agents to the R-CHOP induction backbone, such as bevacizumab [26], obinutuzumab [27], lenalidomide [28], ibrutinib [29], everolimus [30] and enzastaurin [31] have all failed to achieve success despite promising early signals of efficacy. In the REMARC study, maintenance therapy with lenalidomide significantly prolonged progression-free survival (PFS) over placebo, in elderly patients with DLBCL responding to R-CHOP, though no OS benefit was observed [32]. Since the intent of upfront treatment is curative, the value of a PFS benefit without improvement in OS remains contentious, since patients may still be successfully salvaged upon progression. Additionally, given the non-trivial toxicities of lenalidomide in this susceptible population, including high grade neutropenia and cutaneous reactions, the optimal use of this approach remains to be determined.

Polatuzumab vedotin is a CD79b-directed ADC covalently bound to monomethyl auristatin E, a microtubule-disrupting agent. CD79b, a subunit of a heterodimer transmembrane component of the B-cell antigen receptor, is ubiquitously expressed on mature B-cell lymphomas, including DLBCL [33–36]. Recently, polatuzumab vedotin in combination with rituximab, cyclophosphamide, doxorubicin and prednisone (pola-R-CHP) was demonstrated in a placebo-controlled phase III trial to improve PFS over R-CHOP in patients with previously untreated intermediate to high-risk DLBCL (IPI score at least 2) [37, 38]. At two years, the PFS was 76.7% versus 70.2%, respectively. Exploratory subgroup analysis favored pola-R-CHP for patients above 60 years of age, patients with the activated B-cell subtype of DLBCL, absence of bulky disease, and patients who had higher IPI scores between 3 and 5. Importantly, patients in the pola-R-CHP arm received less subsequent treatment (22.5%) as compared to the R-CHOP arm (30.3%), including radiotherapy, systemic therapy, stem-cell transplantation, and CAR-T therapy. In terms of safety profile, grade 3/4 adverse events were comparable in both groups. However, no significant OS difference was observed between both arms. The POLARIX study has since been deemed by the National Institute for Health and Care Excellence (NICE, UK) as the first trial in over 20 years to show meaningful benefit in 20 years over R-CHOP [39], and has similarly been backed by the US Food and Drug Administration (FDA) [40]. Similarly in Singapore, pola-R-CHP has recently received new drug indication approvals by the Health Sciences Authority (HSA), though the regimen is not yet listed on the CDL [41]. Given current cost constraints, both patient preference and disease factors may be taken into consideration for the use of pola-R-CHP in patients with intermediate to high-risk DLBCL.

*Our local practice preferences for standard-risk and intermediate to high-risk DLBCL include:*

1. *For standard-risk DLBCL: R-CHOP chemoimmunotherapy given in six 21-day cycles.*
2. *For intermediate to high-risk DLBCL with an IPI score of 2–5, Pola-R-CHP is currently not reimbursed but can be considered in selected patients where cost constraints are not a limiting factor.*

### Low risk patients with limited-stage disease

DLBCL is localized at the time of diagnosis in 25% to 30% of the patients [42]. In clinical practice, the IPI [38] remains one of the most widely used prognostic models for risk stratification of DLBCL. In limited stage (I or II) disease with aa-IPI of 0 and non-bulky disease (< 7.5 cm), contemporary treatment with six cycles of R-CHOP result in highly favorable outcomes with 6-year PFS approximating 90% and OS at 95% [18, 19].

In the pre-rituximab era, the SWOG 8736 study had showed that in limited stage disease, three cycles of CHOP followed by consolidation radiation therapy was non-inferior to eight cycles of CHOP alone in terms of long term survival outcomes, though either modalities resulted in continuous relapse risk and are inadequate by modern standards [43, 44]. In the rituximab era, several key studies have evaluated the optimal cycle length of systemic R-CHOP therapy, the need for radiation therapy, as well as the potential of a response-adapted approach using positron emission tomography (PET), all of which remain areas of controversy [42, 45]. Of particular note, for patients with “low risk” DLBCL, the phase III FLYER study had examined a cohort of young patients aged 18–60 years diagnosed with limited stage and non-bulky (< 7.5 cm) disease, as well as having good ECOG performance status of 0 to 1, and normal serum lactate dehydrogenase (LDH) levels. The study demonstrated that four cycles of R-CHOP therapy followed by two additional cycles of rituximab was non-inferior to standard six cycles of R-CHOP, providing an abbreviated chemotherapy regimen while sparing radiation for this group of patients with favorable prognosis [46].

A PET response-adapted approach to deciding on the number of chemotherapy courses or delivery of radiation therapy has been evaluated in several studies, and may be particularly useful for selected “low risk” DLBCL cases not meeting FLYER criteria (e.g. elderly patients, or presence of high-risk features). The S1001 phase II study demonstrated excellent survival results in patients (non-bulky < 10 cm, stage I/II untreated DLBCL) treated with four cycles of R-CHOP in the event of a negative interim PET (after cycle 3), which occurred in the majority (89.1%) of all evaluable patients [47]. The LYSA group LNH 2009-1B phase III study investigated previously untreated stage I-II DLBCL patients, aged 18–80 years with aa-IPI scores of 0. In the experimental arm, patients who achieved a negative interim PET (after cycle 2) of R-CHOP (80.4% of patients) went on to receive a total of four cycles, whereas those with an interim positive PET went on to receive a total of six cycles. The 3-year PFS was 92.0% in the experimental arm as compared to 89.2% in the standard arm consisting of six cycles of R-CHOP regardless of interim PET results, demonstrating non-inferiority [48]. Taken together, an abbreviated course of chemoimmunotherapy might be considered for elderly patients with limited stage disease in whom systemic toxicity is a concern (preferably PET-guided if available), while reserving consolidation radiation therapy to those with initial bulky disease or residual disease at the end of treatment. Similarly, abbreviated therapy might be considered for young patients meeting FLYER criteria, or otherwise in those with a negative interim PET, avoiding long term toxicity concerns of radiotherapy.

*Our local practice preferences for low risk DLBCL are variable and include:*

1. *R-CHOP chemoimmunotherapy given in six 21-day cycles as for standard-risk DLBCL.*
2. *Abbreviated R-CHOP for four cycles, with or without 2 additional rituximab doses, if meeting FLYER criteria.*
3. *Combined modality treatment with abbreviated R-CHOP for three cycles followed by consolidation radiation therapy.*
4. *A PET-guided approach with three to four cycles of R-CHOP followed by consideration of consolidation radiation therapy.*

### Double-expressor, double-hit and high risk genetics

The majority of DLBCL cases are of germinal center B-cell (GCB) or activated B-cell (ABC) origin [49–52], and several studies have demonstrated an inferior prognosis for ABC-type DLBCL [53, 54]. While the gene expression-based assays (e.g. Lymph2Cx) [52] are often deployed in clinical trials to differentiate GCB or ABC subtypes, immunohistochemistry-based algorithms (e.g. Han’s criteria) are most frequently used in clinical practice, the latter of which allows the determination into GCB and non-GCB subtypes [53]. Attempts to overcome this poor outcome by addition of novel agents to the R-CHOP backbone, for example ibrutinib [29] or lenalidomide [28] have not been successful despite biological rationale, suggesting a therapeutic approach stratified by cell-of-origin classification may not be that straightforward. It is well known that a high proportion of aggressive B-cell lymphomas harbor high-protein expression of MYC and BCL2 and/or BCL6. These so-called double-expressor DLBCL have been correlated with an inferior outcome following standard therapies [55–60]. The pathogenetic mechanism for the double-expressor phenotype can be broadly divided into those related to, or lacking genomic rearrangements, the latter of which generally belongs to the ABC subtype [61].

Patients with high grade B-cell lymphomas defined by *MYC* and *BCL2* and/or *BCL6* genomic rearrangements, or so-called a double-hit or triple-hit lymphomas, have been well-documented to confer worse prognosis after standard treatment with R-CHOP chemoimmunotherapy [62]. In a more recent retrospective study by the Lunenburg Lymphoma Biomarker Consortium on 2383 patients with DLBCL, *MYC* rearrangements occurred in 11% of the cases and led to worse survival outcomes especially within the first 2 years from diagnosis, with 5-year survival rates of approximately 60% [60]. Importantly, when analysed with respect to the presence of *BCL2* and/or *BCL6* translocations, only cases with double-hit or triple-hit, but not *MYC* rearrangement alone, influenced PFS and OS. In addition, their results suggest that *MYC* rearrangements to a non-immunoglobulin gene partner do not affect survival outcomes.

In terms of frontline induction and consolidation strategies, there is currently no single standard approach for both double-expressor DLBCL and high grade B-cell lymphomas with *MYC* rearrangements, especially for the latter group that are double-hit or triple-hit. Dose-intense regimens [63–66] and consolidation with high dose chemotherapy and stem cell transplant have been explored [21, 67], though their utility remains controversial [68]. In a propensity score-matched retrospective Italian study in patients with double-expressor DLBCL, treatment with either DA-EPOCH-R or R-CHOP showed similar PFS and OS, though younger patients below the age of 65 years achieved a significantly better 2-year PFS with DA-EPOCH-R compared with R-CHOP [69]. Several groups have also adopted DA-EPOCH-R for *MYC*-rearranged DLBCL based on results of a prospective multicentre phase II trial, in which the majority of patients had advanced disease and yet achieving a 48-month EFS and OS of 71% and 77%, respectively [70]. Meta-analysis of 11 retrospective studies showed favorable PFS with this more intensive regimen over R-CHOP, though no difference in OS was observed [71]. Notably however, in the intergroup Alliance/CALGB 50303 phase III, DA-EPOCH-R failed to improve survival outcomes compared with R-CHOP, and was associated with higher toxicities, though the study was not designed to address high-risk DLBCL subgroups [22]. With ongoing controversies surrounding high-grade B-cell lymphoma with *MYC* and *BCL6* rearrangements as a distinct biological and clinical entity [1, 2], as well as the significance of the MYC rearrangement gene partner, the optimal treatment regimen will need to be further defined in prospective clinical trials. Given these uncertainties, R-CHOP remains commonly utilized in this group of high grade B-cell lymphomas harboring high risk genetics.

*Our local practice preferences for DLBCL with high-risk genetics include:*

1. *Single MYC rearrangements: R-CHOP chemoimmunotherapy given in six 21-day cycles as for standard-risk DLBCL; Pola-R-CHP can be considered as for intermediate to high-risk DLBCL with an IPI score of 2–5, in selected patients where cost constraints are not a limiting factor.*
2. *Double-expressor DLBCL: R-CHOP chemoimmunotherapy given in six 21-day cycles as for standard-risk DLBCL; Pola-R-CHP can be considered as for intermediate to high-risk DLBCL with an IPI score of 2–5, in selected patients where cost constraints are not a limiting factor.*
3. *Double-hit or triple-hit: DA-EPOCH-R or R-CHOP given in six 21-day cycles.*

## Special considerations

### Elderly and frail patients

The treatment of DLBCL in elderly patients over 80 years old and in frail patients remains challenging, requiring a fine balance of both maximising therapeutic efficacy and tolerability. Large prospective studies on DLBCL establishing R-CHOP as standard frontline therapy have generally excluded these vulnerable patients who represent a group with poorer prognosis [72–74].

A multicentre, single arm phase II study evaluated the use of rituximab in combination with dose-reduced CHOP (R-miniCHOP) in patients aged over 80 years who had DLBCL and a good performance status, achieving a 2-year PFS of 47% and 2-year OS of 59% [75]. Treatment-related toxicities were generally acceptable, and most of the observed severe haematological and infectious complications, as well as treatment-related deaths occurred during the first cycle of treatment, suggesting the need for prephase treatment with steroids [76]. Recently, the addition of lenalidomide to R-miniCHOP (R2-miniCHOP) was investigated in a phase III study, though it could not demonstrate any significant improvement in OS (2-year OS 66% both arms) [77].

Several smaller studies have also investigated the use of more tolerable regimens over R-CHOP, focusing on this unique patient cohort. In a phase II study, three cycles of abbreviated chemoimmunotherapy either with or without doxorubicin followed by four courses of maintenance rituximab were used to treat elderly patients aged 70 years or older (n = 51; 43% aged 80 years or older) who are poor candidates for standard R-CHOP therapy, achieving 2-year PFS of 71% and 2-year OS of 72% [78]. Another phase II study (n = 14) on rituximab and bendamustine in elderly patients 80 years or deemed ineligible for R-CHOP by physician’s discretion demonstrated overall response rates of 69%, including complete response rates of 54%. The median PFS and OS were both 7.7 months [79]. Chemotherapy-free regimens such as the use of rituximab plus lenalidomide [80] and ibrutinib, rituximab plus lenalidomide [81] have also been suggested to be clinically efficacious and safe in elderly patients.

In patients with underlying comorbidities such as cardiac dysfunction precluding the use of anthracyclines, the substitution of etoposide for doxorubicin (R-CEOP) has been suggested as a potential alternative to R-CHOP, while preserving curative potential. Long term disease-specific survival outcomes at 10 years were not significantly different for R-CEOP and R-CHOP, though OS was lower in the R-CEOP group [82]. On a similar note, substitution of non-pegylated liposomal doxorubicin for doxorubicin (R-COMP) has been proposed as an alternative regimen, though a randomized phase III trial comparing R-COMP to standard R-CHOP in patients with newly diagnosed DLBCL and normal cardiac function failed to demonstrate a significant reduction in cardiotoxicity [83]. A single-arm phase II multicentre trial of rituximab, gemcitabine, cyclophosphamide, vincristine, and prednisolone (R-GCVP) demonstrated encouraging results, with 2-year PFS of 50% and 2-year OS of 56% [84].

*Our local practice preferences for DLBCL in elderly and frail patients include:*

1. *R-miniCHOP chemoimmunotherapy given in six 21-day cycles.*
2. *Other regimens including R-GCVP or R-CVP may be considered, particularly in the setting of cardiac dysfunction.*
3. *For limited stage DLBCL, abbreviated R-CHOP for three to four cycles followed by consideration of consolidation radiation therapy, possibly guided by end of treatment PET imaging.*

### Disease site-specific considerations

Primary mediastinal B-cell lymphoma (PMBCL) is a distinct clinical and biologic entity recognized by the WHO Lymphoma Classification, originating from thymic B-cells [1]. Characterized by a peak incidence in the adolescent and young adult population, female preponderance, mediastinal presentation and unique molecular landscape, there is currently no uniform standard of care, with several strategies derived from both adult and pediatric studies [85]. R-V/MACOP-B (rituximab, etoposide or methotrexate, doxorubicin, cyclophosphamide, vincristine, prednisone, bleomycin) and R-CHOP regimens, typically followed by consolidation radiation therapy, have resulted in favorable outcomes in PMBCL [86, 87]. In addition, DA-EPOCH-R without consolidation radiation therapy has been evaluated in a phase II trial in PMBCL so as to reduce long term toxicity in these young and predominantly female patients [88–90]. Though the outcomes of this study were impressive, with a 5-year EFS of 93%, the optimal systemic therapy and need for consolidation radiation, particularly in the setting of complete metabolic response, remain to be further evaluated. In Singapore, a multicentre study of 124 patients with PMBCL revealed superior survival outcomes for R-CHOP plus radiation and DA-EPOCH-R, when compared with R-CHOP alone—5-year PFS were 90% vs 88.5% vs 56%, respectively [91].

Apart from PMBCL, the optimal treatment strategies of other rare primary extranodal subtypes of DLBCL are less well-defined. In primary breast DLBCL, treatment with R-CHOP chemoimmunotherapy remains an acceptable frontline option, with the role of irradiation and central nervous system (CNS) prophylaxis remaining controversial despite frequent breast and CNS relapses [92–94]. Primary testicular lymphoma represents another unique subtype of large B-cell lymphoma in an “immune-privileged site” [1], and has a tendency to disseminate extranodally including to the contralateral testis and CNS [95, 96]. Orchidectomy followed by R-CHOP in combination with CNS prophylaxis and locoregional radiation therapy has been recommended as first-line treatment, especially for patients with limited stage disease [97, 98]. Other subtypes of large B-cell lymphomas are beyond the scope of this discussion and will not be further elaborated.

*Our local practice preferences for site-specific DLBCL include:*

1. *Primary mediastinal DLBCL: DA-EPOCH-R given in six 21-day cycles; consideration of consolidation radiation therapy in selected patients*
2. *Primary breast DLBCL: R-CHOP chemoimmunotherapy given in four to six 21-day cycles, depending on the presence of risk factors*
3. *Primary testicular: R-CHOP chemoimmunotherapy given in six 21-day cycles, in combination with CNS prophylaxis and locoregional radiation therapy (including the contralateral testis)*
4. *Primary gastrointestinal: R-CHOP chemoimmunotherapy given in four to six 21-day cycles, depending on the presence of risk factors; consideration of dose attenuation in cycle one and prephase treatment*

### DLBCL in the setting of HIV/AIDS

HIV infection and acquired immunodeficiency syndrome (AIDS) is associated with several lymphoproliferative neoplasms including DLBCL [99]. The effectiveness of infusional EPOCH-R and R-CHOP has been evaluated in phase II trials on patients with HIV-associated DLBCL and other high-grade lymphoma [100–102]. A pooled analysis of 150 patients treated on AIDS Malignancy Consortium (AMC) studies of either R-CHOP or EPOCH-R showed that EFS and OS outcomes favored the EPOCH-R regimen [103]. Further results from a large pooled analysis of 1546 patients from 19 prospective clinical trials also showed that the use of rituximab improved survival outcomes and demonstrated greater efficacy for the use of EPOCH as compared to CHOP in HIV-associated lymphoma [104]. While six cycles of EPOCH-R may represent the preferred regimen for HIV-associated DLBCL, early data suggests that an abbreviated treatment course may be sufficient for good risk patients. Dunleavy et al. reported a phase II trial whereby patients with HIV-associated DLBCL could be treated with three cycles of EPOCH-RR (etoposide, prednisone, vincristine, cyclophosphamide, doxorubicin—double dose rituximab) given a negative interim PET after cycle 2 [105]. Similarly, in a post-hoc analysis of a phase II trial conducted by the AIDS Malignancy Consortium, 2-year EFS was similar in patients achieving early complete response after two cycles of EPOCH-R who then went on to complete a total of four cycles, as compared to those who received up to six cycles following documentation of complete response only at cycle 4 [106, 107]. Whether a response-adapted strategy may allow an abbreviated treatment duration while not compromising treatment outcomes remain to be further evaluated in further studies.

*Our local practice preferences for DLBCL in the setting of HIV/AIDS include:*

1. *DA-EPOCH-R given in six 21-day cycles*
2. *Abbreviated three cycles of EPOCH-RR*

## Role of CNS prophylaxis in DLBCL

The topic of CNS prophylaxis remains a highly controversial area in the management of DLBCL. Approximately 5% of patients with DLBCL develop relapse in the CNS despite treatment with chemoimmunotherapy [108]. The CNS International Prognostic Index (CNS-IPI) further enables the identification of patient subgroups at the highest risk of CNS relapse [109]. Criteria for CNS prophylaxis in DLBCL vary amongst consensus guidelines, but generally includes patients with high CNS-IPI scores of 4–6, disease involvement of more than 2 extranodal sites, double-hit or triple-hit genetics, and involvement of high-risk extranodal sites (e.g. testicular, renal/adrenal, breast, bone marrow). There is also ongoing debate regarding the efficacy of intrathecal (IT) and high-dose intravenous methotrexate (HD-MTX) as the optimal methods for CNS prophylaxis. A systematic review of stand-alone IT prophylaxis had shown no reduction in CNS relapse rates [110], while two recent meta-analyses could not provide evidence for a benefit of IT or intravenous therapy [111, 112]. While major consensus guidelines still suggest the use of HD-MTX as the preferred method for CNS prophylaxis over IT therapy, the optimal and safest way to administer it remains contentious. An intercalated strategy allows for early CNS-directed therapy at the risk of delaying systemic therapy due to inadvertent toxicity, while delivering HD-MTX after completion of systemic therapy may represent a more rational approach that prioritizes systemic control [108]. In general, once the indication for CNS prophylaxis has been identified, HD-MTX is the preferred modality over IT MTX, and an intercalated approach should only be considered if induction systemic therapy would not be compromised.

In Singapore, a previous retrospective study on 499 patients diagnosed with DLBCL and treated with CHOP or R-CHOP showed that poor performance status (ECOG > 1), non-complete response to systemic therapy, and involvement of testis, kidney and breast were independent predictors of CNS relapse. Importantly, IT prophylaxis did not reduce CNS relapse [113]. More recently, a multicentre retrospective study was conducted on patients with DLBCL treated with R-CHOP at three academic medical centers in Singapore. In patients with high CNS-IPI scores of 4–6 or with involvement of high-risk extranodal sites (breast, testis, kidney/adrenal), CNS prophylaxis with HD-MTX of at least 1 g/m2 either intercalated with R-CHOP or given at the end of six cycles, was demonstrated to lower the risk of isolated CNS relapse. However, this did not influence concomitant CNS-systemic relapse rates, systemic relapse rates, or improve survival outcomes [114]. Primary CNS lymphoma is not included within the scope of this discussion.

*Our local practice preferences for CNS prophylaxis in DLBCL are variable and include:*

1. *HD-MTX (at least 1 g/m* ^*2*^ *) for at least 2 cycles, given in an intercalated approach or at the end of induction chemoimmunotherapy*
2. *HD-MTX generally preferred over IT prophylaxis*
3. *Preferred indications include high-risk extranodal site, in particular testicular and renal/adrenal involvement*

## Relapsed or refractory DLBCL

Approximately 30–40% of DLBCL are refractory or relapse despite frontline chemoimmunotherapy [115]. In the second-line setting, high dose chemotherapy followed by ASCT remains one of the curative treatment options, though about two-thirds of patients will continue to relapse [3, 4, 116]. In particular, patients who are refractory or relapse within 1 year from frontline therapy represent a population with dismal outcomes.

Three CD19-directed CAR-T therapies—axicabtagene ciloleucel [117–119], tisagenlecleucel [120–122] and lisocabtagene maraleucel [123] have been approved for adult patients who have relapsed or refractory large B-cell lymphoma including DLBCL who had received 2 or more lines of systemic therapy. Recently, two randomized phase III trials have also shown the superiority of CAR-T-cell products over ASCT in patients with relapsed or refractory disease within 1 year after first-line treatment [124, 125]. The pivotal ZUMA-7 trial in particular reported an overall survival benefit of axicabtagene ciloleucel over standard chemoimmunotherapy followed by high-dose chemotherapy with autologous stem-cell transplantation. The four-year overall survival rate was 54.6% with axicabtagene ciloleucel and 46% with standard care [6].

Other approved therapeutic options include polatuzumab vedotin in combination with bendamustine and rituximab (pola-BR) [126, 127], tafasitamab in combination with lenalidomide [128], loncastuximab tesirine [129] and selinexor [130] which are potentially useful for patients ineligible for ASCT or CAR-T therapy (Table 2). The results of a recent phase II study in the second line treatment of transplant-eligible relapsed aggressive B-cell lymphoma using polatuzumab in combination with R-ICE (pola-R-ICE) has been reported in abstract form. An encouraging response rate of 89% and complete response rate of 61% was achieved, with approximately half the patients proceeding onto ASCT at the time of reporting [131].

**Table 2:** Recent pivotal clinical trials for relapsed/refractory DLBCL

Regimen (Trial name)	Trial design, n	Key inclusion criteria	Major efficacy endpoints
Axicabtagene ciloleucel (ZUMA-1) [117–119]	Phase II 111	Refractory DLBCL—defined as progressive or stable disease as the best response to the most recent chemotherapy regimen or disease progression or relapse within 12 months after ASCT	ORR 82% (CR 49%) Median OS 25.8 months Median PFS 5.9 months
Tisagenlecleucel (JULIET) [120–122]	Phase II 115	Relapsed or refractory DLBCL who were ineligible for or had disease progression after ASCT	ORR 53% (CR 39%) Median OS 11.1 months Median PFS 2.9 months
Lisocabtagene maraleucel (TRANSCEND NHL 001) [123]	Phase II 269	Relapsed or refractory DLBCL after ≥ 2 lines of therapy	ORR 73% (CR 53%) Median OS 21.1 months Median PFS 6.8 months
Axicabtagene ciloleucel (ZUMA-7) [6, 124]	Phase III 359	Refractory to or relapsed ≤ 12 months after first-line treatment	ORR 83% (CR 65%) Median OS not reached Median PFS 14.7 months
Lisocabtagene maraleucel (TRANSFORM) [125]	Phase III 184	Refractory to or relapsed ≤ 12 months after first-line treatment	ORR 86% (CR 66%) Median OS not reached Median PFS 14.8 months
Polatuzumab vedotin, bendamustine, rituximab [126, 127]	Phase II 80	Relapsed or refractory DLBCL after ≥ 1 line of therapy and were transplant-ineligible	ORR 52.5% (CR 40%) Median OS 12.4 months Median PFS 9.5 months
Tafasitamab, lenalidomide (L-MIND) [128]	Phase II 80	Relapsed or refractory DLBCL after ≥ 1 line of therapy and were transplant-ineligible	ORR 60% (CR 43%) Median OS not reached Median PFS 12.1 months
Loncastuximab tesirine (LOTIS-2) [129]	Phase II 145	Relapsed or refractory DLBCL after ≥ 2 lines of therapy	ORR 48% (CR 24%) Median OS 9.9 months Median PFS 4.9 months
Selinexor (SADAL) [130]	Phase II 127	Relapsed or refractory DLBCL after ≥ 2 lines of therapy and progressed after or were transplant-ineligible	ORR 28% (CR 12%) Median OS 9.1 months Median PFS 2.6 months

*ORR* objective response rate, *CR* complete response, *PR* partial response, *OS* overall survival, *PFS* progression-free survival, *DOR* duration of response, *ASCT* autologous stem cell transplantation

With the current armamentarium available, it has become increasing challenging to decide on the optimal therapy after failure of frontline treatment. Apart from efficacy and safety concerns, the financial burden that these new treatment modalities place on the patient and larger healthcare system presents as an important issue as well. And even as we are learning how to navigate the rapidly evolving therapeutic landscape of DLBCL, emerging novel strategies such as bispecific antibodies glofitamab [132], epcoritamab [133, 134] and odronextamab [135] are already expected to add to the complexity of managing patients with DLBCL in the immediate future. The US FDA has just granted approval for glofitamab and epcoritamab for the treatment of patients with relapsed/refractory DLBCL after two or more lines of systemic therapy [136, 137]. A challenging question that remains to be answered will be the optimal sequence in which to use these novel therapeutic modalities, and the best combination approach.

*Our local practice preferences for relapsed or refractory DLBCL include:*

1. *For transplant eligible: R-ICE, R-DHAP, R-GDP, Pola-R-ICE followed by ASCT*
2. *For transplant ineligible: R-GDP, R-GEMOX, Pola-BR, Pola-R-ICE*
3. *CAR-T therapy is available for indicated use though not currently reimbursed in Singapore, and can be considered in selected patients where cost constraints are not a limiting factor.*

## Next generation sequencing in the clinic—ready for primetime?

Our understanding of the molecular landscape of DLBCL has improved significantly over the past decade, highlighting unique genomic subtypes with implications on targeted therapy [138, 139]. These molecular classification systems are anticipated to enable new trials for targeted therapies, with the potential for clinical implementation. This builds upon earlier incorporations of genomic information into the modern framework for DLBCL sub-classification, based on cell-of-origin by gene transcription profiling, and gene rearrangements of *MYC*, *BCL2* and/or *BCL6*. Chapuy et al. analysed 304 DLBCL cases and identified five genetic clusters with distinct signatures termed C1 to C5 [140], while the National Cancer Institute (NCI) group examined 574 cases to identify seven genetic subtypes termed BN2, A53, EZB (MYC + and MYC-), ST2, MCD, and N1 [141, 142]. Both these classification systems are based on whole exome and deep targeted sequencing, and share significant overlap—C1 or BN2 harbors *BCL6* rearrangements and *NOTCH2* mutations; C2 or A53 carries *TP53* alterations and aneuploidy; C3 or EZB is characterized by *BCL2* translocations and mutations in *CREBBP*, *EZH2* and *KMT2D*; C4 or ST2 carries mutations in *SGK1*; C5 or MCD carries mutations in *MYD88* and *CD79B*; N1 carries *NOTCH1* mutations. The Haematological Malignancy Research Network (HMRN) further validated the reproducibility of these molecular subtypes and their potential clinical applicability in a larger cohort of 928 DLBCL formalin-fixed paraffin-embedded (FFPE) samples using a 293-gene targeted sequencing panel, demonstrating the recapitulation of major subtypes [143]. Notably, the NCI classification has now been developed into a publically-available LymphGen probabilistic classifier that is able to assign individual patients to one of these seven genetic subtypes.

Recent data suggests that the molecular heterogeneity of DLBCL may provide a rational explanation for the failure of previous targeted therapy trials. In the PHOENIX study, patients with non-GCB DLBCL did not benefit from the addition of ibrutinib to R-CHOP therapy [29]. However, younger patients with MCD and N1 DLBCL subtypes had a superior 3-year EFS of 100% with ibrutinib plus R-CHOP, as compared to 42.9% and 50%, respectively, with R-CHOP alone [144]. Though this result is based on retrospective analysis and requires prospective validation, it provides an initial demonstration of the potential for incorporating a molecular consensus based-approach in the design of future clinical studies. In current clinical practice, the value for routine genomic profiling in the frontline or relapsed setting remains unclear [145]. Logistical challenges and cost issues aside, the implications of molecularly subtyping DLBCL on treatment selection are not yet certain. Furthermore, a significant proportion of DLBCL remains unclassifiable [140–143], and the influence of ethnicity and age needs to be further clarified. Perhaps a deeper characterization of the molecular landscape of DLBCL is still warranted, integrating pan-omic data encompassing the genome, transcriptome, epigenome, proteome and metagenome, as well as multi-dimensional information of the tumor microenvironment and host immune system through single cell and spatiotemporal profiling [146].

*Our local practice preferences for DLBCL are currently not determined by molecular consensus classifications.*

## Conclusions

The diagnostic algorithm of DLBCL has evolved over time, building on the foundations of anatomical pathology assessment of tissue architecture and cytomorphology, to the incorporation of immunophenotype, as well as assessment of genetic rearrangements. In the new era of molecular medicine and precision oncology, the diagnosis of DLBCL has and will continue to be refined in the context of clinical relevance and application. Simultaneously, the rapidly growing therapeutic landscape of DLBCL poses a significant challenge to the practicing hemato-oncologist, particularly in having to tackle issues of drug accessibility and cost deliberations in context of the larger socio-economy, all while balancing against the needs and preferences of the individual, in the spirit of personalized care for patients with DLBCL.

## Data Availability

Not applicable.

## References

[1] Alaggio R, Amador C, Anagnostopoulos I (2022). The 5th edition of the World Health Organization classification of haematolymphoid tumours: lymphoid neoplasms. Leukemia.

[2] Campo E, Jaffe ES, Cook JR (2022). The international consensus classification of mature lymphoid neoplasms: a report from the clinical advisory committee. Blood.

[3] Philip T, Guglielmi C, Hagenbeek A (1995). Autologous bone marrow transplantation as compared with salvage chemotherapy in relapses of chemotherapy-sensitive non-Hodgkin's lymphoma. N Engl J Med.

[4] Gisselbrecht C, Glass B, Mounier N et al. Salvage regimens with autologous transplantation for relapsed large B-cell lymphoma in the rituximab era. J Clin Oncol. 2010 Sep 20; 28(27): 4184–90. doi: 10.1200/JCO.2010.28.1618. Epub 2010 Jul 26. Erratum in: J Clin Oncol. 2012 May 20; 30(15): 1896. PMID: 20660832; PMCID: PMC3664033. 10.1200/JCO.2010.28.1618 PMC3664033 20660832

[5] Crump M, Neelapu SS, Farooq U et al. Outcomes in refractory diffuse large B-cell lymphoma: results from the international SCHOLAR-1 study. Blood. 2017 Oct 19; 130(16): 1800–1808. doi: 10.1182/blood-2017-03-769620. Epub 2017 Aug 3. Erratum in: Blood. 2018 Feb 1; 131(5): 587–588. PMID: 28774879; PMCID: PMC5649550. 10.1182/blood-2017-03-769620 PMC5649550 28774879

[6] Westin JR, Oluwole OO, Kersten MJ (2023). Survival with axicabtagene ciloleucel in Large B-cell lymphoma. N Engl J Med.

[7] Nastoupil LJ, Bartlett NL (2023). Navigating the evolving treatment landscape of diffuse large B-cell lymphoma. J Clin Oncol.

[8] Sehn LH, Salles G (2021). Diffuse large B-cell lymphoma. N Engl J Med.

[9] Poletto S, Novo M, Paruzzo L, Frascione PMM, Vitolo U (2022). Treatment strategies for patients with diffuse large B-cell lymphoma. Cancer Treat Rev.

[10] Singapore Cancer Registry Annual Report. 2020. https://nrdo.gov.sg/publications/cancer. Accessed 1 Apr 2023.

[11] Sung H, Ferlay J, Siegel RL (2021). Global Cancer Statistics 2020: GLOBOCAN estimates of incidence and mortality worldwide for 36 cancers in 185 Countries. CA Cancer J Clin.

[12] Lim RB, Loy EY, Lim GH, Zheng H, Chow KY, Lim ST (2015). Gender and ethnic differences in incidence and survival of lymphoid neoplasm subtypes in an Asian population: secular trends of a population-based cancer registry from 1998 to 2012. Int J Cancer.

[13] Ngo L, Hee SW, Lim LC (2008). Prognostic factors in patients with diffuse large B cell lymphoma: before and after the introduction of rituximab. Leuk Lymphoma.

[14] Lim RMH, Chan NPX, Khoo LP (2020). A clinico-genotypic prognostic index for de novo composite diffuse large B-cell lymphoma arising from follicular lymphoma in asian patients treated in the rituximab era. Sci Rep.

[15] Cancer Drug List. https://www.moh.gov.sg/home/our-healthcare-system/medishield-life/what-is-medishield-life/what-medishield-life-benefits/cancer-drug-list. Accessed 1 Apr 2023.

[16] Coiffier B, Lepage E, Briere J, Herbrecht R, Tilly H, Bouabdallah R, Morel P, Van Den Neste E, Salles G, Gaulard P, Reyes F, Lederlin P, Gisselbrecht C (2002). CHOP chemotherapy plus rituximab compared with CHOP alone in elderly patients with diffuse large-B-cell lymphoma. N Engl J Med.

[17] Coiffier B, Thieblemont C, Van Den Neste E (2010). Long-term outcome of patients in the LNH-985 trial, the first randomized study comparing rituximab-CHOP to standard CHOP chemotherapy in DLBCL patients: a study by the Groupe d'Etudes des Lymphomes de l'Adulte. Blood.

[18] Pfreundschuh M, Trümper L, Osterborg A, Pettengell R, Trneny M, Imrie K, Ma D, Gill D, Walewski J, Zinzani PL, Stahel R, Kvaloy S, Shpilberg O, Jaeger U, Hansen M, Lehtinen T, López-Guillermo A, Corrado C, Scheliga A, Milpied N, Mendila M, Rashford M, Kuhnt E, Loeffler M, MabThera International Trial Group (2006). CHOP-like chemotherapy plus rituximab versus CHOP-like chemotherapy alone in young patients with good-prognosis diffuse large-B-cell lymphoma: a randomised controlled trial by the MabThera International Trial (MInT) Group. Lancet Oncol.

[19] Pfreundschuh M, Kuhnt E, Trümper L (2011). CHOP-like chemotherapy with or without rituximab in young patients with good-prognosis diffuse large-B-cell lymphoma: 6-year results of an open-label randomised study of the MabThera International Trial (MInT) Group. Lancet Oncol.

[20] Pfreundschuh M, Schubert J, Ziepert M (2008). Six versus eight cycles of bi-weekly CHOP-14 with or without rituximab in elderly patients with aggressive CD20+ B-cell lymphomas: a randomised controlled trial (RICOVER-60). Lancet Oncol.

[21] Récher C, Coiffier B, Haioun C (2011). Intensified chemotherapy with ACVBP plus rituximab versus standard CHOP plus rituximab for the treatment of diffuse large B-cell lymphoma (LNH03-2B): an open-label randomised phase 3 trial. Lancet.

[22] Bartlett NL, Wilson WH, Jung SH (2019). Dose-adjusted EPOCH-R compared with R-CHOP as frontline therapy for diffuse large B-Cell lymphoma: clinical outcomes of the phase III intergroup trial alliance/CALGB 50303. J Clin Oncol.

[23] Chiappella A, Martelli M, Angelucci E (2017). Rituximab-dose-dense chemotherapy with or without high-dose chemotherapy plus autologous stem-cell transplantation in high-risk diffuse large B-cell lymphoma (DLCL04): final results of a multicentre, open-label, randomised, controlled, phase 3 study. Lancet Oncol.

[24] Schmitz N, Nickelsen M, Ziepert M (2012). Conventional chemotherapy (CHOEP-14) with rituximab or high-dose chemotherapy (MegaCHOEP) with rituximab for young, high-risk patients with aggressive B-cell lymphoma: an open-label, randomised, phase 3 trial (DSHNHL 2002–1). Lancet Oncol.

[25] Stiff PJ, Unger JM, Cook JR (2013). Autologous transplantation as consolidation for aggressive non-Hodgkin's lymphoma. N Engl J Med.

[26] Seymour JF, Pfreundschuh M, Trnĕný M (2014). R-CHOP with or without bevacizumab in patients with previously untreated diffuse large B-cell lymphoma: final MAIN study outcomes. Haematologica.

[27] Vitolo U, Trněný M, Belada D (2017). Obinutuzumab or rituximab plus cyclophosphamide, doxorubicin, vincristine, and prednisone in previously untreated diffuse large B-Cell lymphoma. J Clin Oncol.

[28] Nowakowski GS, Chiappella A, Gascoyne RD (2021). ROBUST: a phase III study of lenalidomide plus R-CHOP versus placebo plus R-CHOP in previously untreated patients with ABC-type diffuse large B-cell lymphoma. J Clin Oncol.

[29] Younes A, Sehn LH, Johnson P (2019). Randomized phase III trial of ibrutinib and rituximab plus cyclophosphamide, doxorubicin, vincristine, and prednisone in non-germinal center B-cell diffuse large B-Cell lymphoma. J Clin Oncol.

[30] Witzig TE, Tobinai K, Rigacci L (2018). Adjuvant everolimus in high-risk diffuse large B-cell lymphoma: final results from the PILLAR-2 randomized phase III trial. Ann Oncol.

[31] Crump M, Leppä S, Fayad L (2016). Randomized, double-blind, phase III trial of enzastaurin versus placebo in patients achieving remission after first-line therapy for high-risk diffuse large B-cell lymphoma. J Clin Oncol.

[32] Thieblemont C, Tilly H, Gomes da Silva M (2017). Lenalidomide maintenance compared with placebo in responding elderly patients with diffuse large b-cell lymphoma treated with first-line rituximab plus cyclophosphamide, doxorubicin, vincristine, and prednisone. J Clin Oncol.

[33] Dornan D, Bennett F, Chen Y (2009). Therapeutic potential of an anti-CD79b antibody-drug conjugate, anti-CD79b-vc-MMAE, for the treatment of non-Hodgkin lymphoma. Blood.

[34] Pfeifer M, Zheng B, Erdmann T (2015). Anti-CD22 and anti-CD79B antibody drug conjugates are active in different molecular diffuse large B-cell lymphoma subtypes. Leukemia.

[35] Okazaki M, Luo Y, Han T, Yoshida M, Seon BK (1993). Three new monoclonal antibodies that define a unique antigen associated with prolymphocytic leukemia/non-Hodgkin's lymphoma and are effectively internalized after binding to the cell surface antigen. Blood.

[36] Polson AG, Yu SF, Elkins K (2007). Antibody-drug conjugates targeted to CD79 for the treatment of non-Hodgkin lymphoma. Blood.

[37] Tilly H, Morschhauser F, Sehn LH (2022). Polatuzumab vedotin in previously untreated diffuse large B-cell lymphoma. N Engl J Med.

[38] Ziepert M, Hasenclever D, Kuhnt E et al. Standard International prognostic index remains a valid predictor of outcome for patients with aggressive CD20+ B-cell lymphoma in the rituximab era. J Clin Oncol. 2010 May 10; 28(14): 2373–80. doi: 10.1200/JCO.2009.26.2493. Epub 2010 Apr 12. Erratum in: J Clin Oncol. 2011 Feb 20; 29(6): 779. PMID: 20385988. 10.1200/JCO.2009.26.2493 20385988

[39] Polatuzumab vedotin in combination for untreated diffuse large B-cell lymphoma Published: 01 March 2023. https://www.nice.org.uk/guidance/ta874. Accessed 4 Apr 2023. 40080621

[40] Oncologic Drugs Advisory Committee (ODAC) Meeting. FDA website. March 9, 2023. https://www.fda.gov/advisory-committees/advisory-committee-calendar/march-9-2023-meeting-oncologic-drugs-advisory-committee-meeting-announcement-03092023. Accessed 4 Apr 2023.

[41] New drug indication approval - January 2023 Published: 31 Jan 2023. https://www.hsa.gov.sg/announcements/new-drug-indication-approvals/new-drug-indication-approval---january-2023. Accessed 4 Apr 2023.

[42] Hawkes EA, Barraclough A, Sehn LH (2022). Limited-stage diffuse large B-cell lymphoma. Blood.

[43] Miller TP, Dahlberg S, Cassady JR (1998). Chemotherapy alone compared with chemotherapy plus radiotherapy for localized intermediate- and high-grade non-Hodgkin's lymphoma. N Engl J Med.

[44] Stephens DM, Li H, LeBlanc ML (2016). Continued risk of relapse independent of treatment modality in limited-stage diffuse large B-cell lymphoma: final and long-term analysis of southwest oncology group study S8736. J Clin Oncol.

[45] Rojek AE, Smith SM (2022). Evolution of therapy for limited stage diffuse large B-cell lymphoma. Blood Cancer J.

[46] Poeschel V, Held G, Ziepert M (2019). Four versus six cycles of CHOP chemotherapy in combination with six applications of rituximab in patients with aggressive B-cell lymphoma with favourable prognosis (FLYER): a randomised, phase 3, non-inferiority trial. Lancet.

[47] Persky DO, Li H, Stephens DM et al. positron emission tomography-directed therapy for patients with limited-stage diffuse large B-cell lymphoma: results of intergroup national clinical trials network study S1001. J Clin Oncol. 2020 Sep 10; 38(26): 3003–3011. doi: 10.1200/JCO.20.00999. Epub 2020 Jul 13. Erratum in: J Clin Oncol. 2020 Oct 10; 38(29): 3459. PMID: 32658627; PMCID: PMC7479758. 10.1200/JCO.20.00999 PMC7479758 32658627

[48] Bologna S (2021). Early positron emission tomography response-adapted treatment in localized diffuse large B-cell lymphoma (aaIPI=0): results of the phase 3 LYSA LNH 09–1B trial. Hematol Oncol.

[49] Alizadeh AA, Eisen MB, Davis RE (2000). Distinct types of diffuse large B-cell lymphoma identified by gene expression profiling. Nature.

[50] Rosenwald A, Wright G, Chan WC (2002). The use of molecular profiling to predict survival after chemotherapy for diffuse large-B-cell lymphoma. N Engl J Med.

[51] Lenz G, Staudt LM (2010). Aggressive lymphomas. N Engl J Med.

[52] Scott DW, Mottok A, Ennishi D (2015). Prognostic significance of diffuse large B-Cell lymphoma cell of origin determined by digital gene expression in formalin-fixed paraffin-embedded tissue biopsies. J Clin Oncol.

[53] Hans CP, Weisenburger DD, Greiner TC (2004). Confirmation of the molecular classification of diffuse large B-cell lymphoma by immunohistochemistry using a tissue microarray. Blood.

[54] Meyer PN, Fu K, Greiner TC (2011). Immunohistochemical methods for predicting cell of origin and survival in patients with diffuse large B-cell lymphoma treated with rituximab. J Clin Oncol.

[55] Johnson NA, Slack GW, Savage KJ (2012). Concurrent expression of MYC and BCL2 in diffuse large B-cell lymphoma treated with rituximab plus cyclophosphamide, doxorubicin, vincristine, and prednisone. J Clin Oncol.

[56] Hu S, Xu-Monette ZY, Tzankov A (2013). MYC/BCL2 protein coexpression contributes to the inferior survival of activated B-cell subtype of diffuse large B-cell lymphoma and demonstrates high-risk gene expression signatures: a report from The International DLBCL Rituximab-CHOP Consortium Program. Blood.

[57] Perry AM, Alvarado-Bernal Y, Laurini JA (2014). MYC and BCL2 protein expression predicts survival in patients with diffuse large B-cell lymphoma treated with rituximab. Br J Haematol.

[58] Herrera AF, Mei M, Low L (2017). Relapsed or refractory double-expressor and double-hit lymphomas have inferior progression-free survival after autologous stem-cell transplantation. J Clin Oncol.

[59] Savage KJ, Slack GW, Mottok A (2016). Impact of dual expression of MYC and BCL2 by immunohistochemistry on the risk of CNS relapse in DLBCL. Blood.

[60] Rosenwald A, Bens S, Advani R (2019). Prognostic significance of MYC rearrangement and translocation partner in diffuse large B-cell lymphoma: a study by the lunenburg lymphoma biomarker consortium. J Clin Oncol.

[61] Scott DW, King RL, Staiger AM (2018). High-grade B-cell lymphoma with *MYC* and *BCL2* and/or *BCL6* rearrangements with diffuse large B-cell lymphoma morphology. Blood.

[62] Petrich AM, Gandhi M, Jovanovic B (2014). Impact of induction regimen and stem cell transplantation on outcomes in double-hit lymphoma: a multicenter retrospective analysis. Blood.

[63] Chamuleau MED, Burggraaff CN, Nijland M (2020). Treatment of patients with MYC rearrangement positive large B-cell lymphoma with R-CHOP plus lenalidomide: results of a multicenter HOVON phase II trial. Haematologica.

[64] Leppä S, Jørgensen J, Tierens A (2020). Patients with high-risk DLBCL benefit from dose-dense immunochemotherapy combined with early systemic CNS prophylaxis. Blood Adv.

[65] McMillan AK, Phillips EH, Kirkwood AA (2020). Favourable outcomes for high-risk diffuse large B-cell lymphoma (IPI 3–5) treated with front-line R-CODOX-M/R-IVAC chemotherapy: results of a phase 2 UK NCRI trial. Ann Oncol.

[66] Laude MC, Lebras L, Sesques P (2021). First-line treatment of double-hit and triple-hit lymphomas: survival and tolerance data from a retrospective multicenter French study. Am J Hematol.

[67] Molina TJ, Canioni D, Copie-Bergman C (2014). Young patients with non-germinal center B-cell-like diffuse large B-cell lymphoma benefit from intensified chemotherapy with ACVBP plus rituximab compared with CHOP plus rituximab: analysis of data from the Groupe d'Etudes des Lymphomes de l'Adulte/lymphoma study association phase III trial LNH 03–2B. J Clin Oncol.

[68] Dunleavy K (2021). Double-hit lymphoma: optimizing therapy. Hematol Am Soc Hematol Educ Program.

[69] Dodero A, Guidetti A, Tucci A (2019). Dose-adjusted EPOCH plus rituximab improves the clinical outcome of young patients affected by double expressor diffuse large B-cell lymphoma. Leukemia.

[70] Dunleavy K, Fanale MA, Abramson JS (2018). Dose-adjusted EPOCH-R (etoposide, prednisone, vincristine, cyclophosphamide, doxorubicin, and rituximab) in untreated aggressive diffuse large B-cell lymphoma with MYC rearrangement: a prospective, multicentre, single-arm phase 2 study. Lancet Haematol.

[71] Howlett C, Snedecor SJ, Landsburg DJ (2015). Front-line, dose-escalated immunochemotherapy is associated with a significant progression-free survival advantage in patients with double-hit lymphomas: a systematic review and meta-analysis. Br J Haematol.

[72] Habermann TM, Weller EA, Morrison VA (2006). Rituximab-CHOP versus CHOP alone or with maintenance rituximab in older patients with diffuse large B-cell lymphoma. J Clin Oncol.

[73] Thieblemont C, Grossoeuvre A, Houot R (2008). Non-Hodgkin's lymphoma in very elderly patients over 80 years. A descriptive analysis of clinical presentation and outcome. Ann Oncol.

[74] Advani RH, Chen H, Habermann TM (2010). Comparison of conventional prognostic indices in patients older than 60 years with diffuse large B-cell lymphoma treated with R-CHOP in the US Intergroup Study (ECOG 4494, CALGB 9793): consideration of age greater than 70 years in an elderly prognostic index (E-IPI). Br J Haematol.

[75] Peyrade F, Jardin F, Thieblemont C (2011). Attenuated immunochemotherapy regimen (R-miniCHOP) in elderly patients older than 80 years with diffuse large B-cell lymphoma: a multicentre, single-arm, phase 2 trial. Lancet Oncol.

[76] Peyrade F, Bologna S, Delwail V (2017). Combination of ofatumumab and reduced-dose CHOP for diffuse large B-cell lymphomas in patients aged 80 years or older: an open-label, multicentre, single-arm, phase 2 trial from the LYSA group. Lancet Haematol.

[77] Oberic L, Peyrade F, Puyade M (2021). Subcutaneous Rituximab-MiniCHOP compared with subcutaneous Rituximab-MiniCHOP plus lenalidomide in diffuse large B-cell lymphoma for patients age 80 years or older. J Clin Oncol.

[78] Hainsworth JD, Flinn IW, Spigel DR (2010). Brief-duration rituximab/chemotherapy followed by maintenance rituximab in patients with diffuse large B-cell lymphoma who are poor candidates for R-CHOP chemotherapy: a phase II trial of the Sarah Cannon Oncology Research Consortium. Clin Lymphoma Myeloma Leuk.

[79] Weidmann E, Neumann A, Fauth F (2011). Phase II study of bendamustine in combination with rituximab as first-line treatment in patients 80 years or older with aggressive B-cell lymphomas. Ann Oncol.

[80] Zinzani PL, Pellegrini C, Gandolfi L, Stefoni V, Quirini F, Derenzini E, Broccoli A, Argnani L, Pileri S, Baccarani M (2011). Combination of lenalidomide and rituximab in elderly patients with relapsed or refractory diffuse large B-cell lymphoma: a phase 2 trial. Clin Lymphoma Myeloma Leuk.

[81] Xu PP, Shi ZY, Qian Y, Cheng S, Zhu Y, Jiang L, Li JF, Fang H, Huang HY, Yi HM, Ouyang BS, Wang L, Zhao WL (2022). Ibrutinib, rituximab, and lenalidomide in unfit or frail patients aged 75 years or older with de novo diffuse large B-cell lymphoma: a phase 2, single-arm study. Lancet Healthy Longev.

[82] Moccia AA, Schaff K, Freeman C (2021). Long-term outcomes of R-CEOP show curative potential in patients with DLBCL and a contraindication to anthracyclines. Blood Adv.

[83] Fridrik MA, Jaeger U, Petzer A (2016). Cardiotoxicity with rituximab, cyclophosphamide, non-pegylated liposomal doxorubicin, vincristine and prednisolone compared to rituximab, cyclophosphamide, doxorubicin, vincristine, and prednisolone in frontline treatment of patients with diffuse large B-cell lymphoma: a randomised phase-III study from the Austrian Cancer Drug Therapy Working Group [Arbeitsgemeinschaft Medikamentöse Tumortherapie AGMT](NHL-14). Eur J Cancer.

[84] Fields PA, Townsend W, Webb A et al. De novo treatment of diffuse large B-cell lymphoma with rituximab, cyclophosphamide, vincristine, gemcitabine, and prednisolone in patients with cardiac comorbidity: a United Kingdom National Cancer Research Institute trial. J Clin Oncol. 2014 Feb 1; 32(4): 282–7. doi: 10.1200/JCO.2013.49.7586. Epub 2013 Nov 12. Erratum in: J Clin Oncol. 2014 Oct 20; 32(30): 3461. PMID: 24220559. 10.1200/JCO.2013.49.7586 24220559

[85] Giulino-Roth L (2018). How I treat primary mediastinal B-cell lymphoma. Blood.

[86] Savage KJ, Al-Rajhi N, Voss N (2006). Favorable outcome of primary mediastinal large B-cell lymphoma in a single institution: the British Columbia experience. Ann Oncol.

[87] Rieger M, Osterborg A, Pettengell R (2011). Primary mediastinal B-cell lymphoma treated with CHOP-like chemotherapy with or without rituximab: results of the Mabthera International Trial Group study. Ann Oncol.

[88] Dunleavy K, Pittaluga S, Maeda LS (2013). Dose-adjusted EPOCH-rituximab therapy in primary mediastinal B-cell lymphoma. N Engl J Med.

[89] Giulino-Roth L, O'Donohue T, Chen Z (2017). Outcomes of adults and children with primary mediastinal B-cell lymphoma treated with dose-adjusted EPOCH-R. Br J Haematol.

[90] Shah NN, Szabo A, Huntington SF et al. R-CHOP versus dose-adjusted R-EPOCH in frontline management of primary mediastinal B-cell lymphoma: a multi-centre analysis. Br J Haematol. 2018 Feb; 180(4): 534–544. doi: 10.1111/bjh.15051. Epub 2017 Dec 19. Erratum in: Br J Haematol. 2018 Apr; 181(1): 152. PMID: 29265182. 10.1111/bjh.15051 29265182

[91] Chan EHL, Koh LP, Lee J (2019). Real world experience of R-CHOP with or without consolidative radiotherapy vs DA-EPOCH-R in the first-line treatment of primary mediastinal B-cell lymphoma. Cancer Med.

[92] Aviv A, Tadmor T, Polliack A (2013). Primary diffuse large B-cell lymphoma of the breast: looking at pathogenesis, clinical issues and therapeutic options. Ann Oncol.

[93] Hu S, Song Y, Sun X et al. Primary breast diffuse large B-cell lymphoma in the rituximab era: Therapeutic strategies and patterns of failure. Cancer Sci. 2018 Dec; 109(12): 3943–3952. doi: 10.1111/cas.13828. Epub 2018 Nov 11. Erratum in: Cancer Sci. 2019 Apr; 110(4): 1503. PMID: 30302857; PMCID: PMC6272095. 10.1111/cas.13828 PMC6272095 30302857

[94] Yhim HY, Yoon DH, Kim SJ (2020). First-line treatment for primary breast diffuse large b-cell lymphoma using immunochemotherapy and central nervous system prophylaxis: a multicenter phase 2 trial. Cancers (Basel).

[95] Cheah CY, Wirth A, Seymour JF (2014). Primary testicular lymphoma. Blood.

[96] Deng L, Xu-Monette ZY, Loghavi S (2016). Primary testicular diffuse large B-cell lymphoma displays distinct clinical and biological features for treatment failure in rituximab era: a report from the International PTL Consortium. Leukemia.

[97] Vitolo U, Ferreri AJ, Zucca E (2008). Primary testicular lymphoma. Crit Rev Oncol Hematol.

[98] Vitolo U, Chiappella A, Ferreri AJ (2011). First-line treatment for primary testicular diffuse large B-cell lymphoma with rituximab-CHOP, CNS prophylaxis, and contralateral testis irradiation: final results of an international phase II trial. J Clin Oncol.

[99] Little RF, Dunleavy K (2013). Update on the treatment of HIV-associated hematologic malignancies. Hematol Am Soc Hematol Educ Program.

[100] Ribera JM, Oriol A, Morgades M (2008). Safety and efficacy of cyclophosphamide, adriamycin, vincristine, prednisone and rituximab in patients with human immunodeficiency virus-associated diffuse large B-cell lymphoma: results of a phase II trial. Br J Haematol.

[101] Dunleavy K, Pittaluga S, Shovlin M (2013). Low-intensity therapy in adults with Burkitt's lymphoma. N Engl J Med.

[102] Roschewski M, Dunleavy K, Abramson JS (2020). Multicenter study of risk-adapted therapy with dose-adjusted EPOCH-R in adults with untreated Burkitt lymphoma. J Clin Oncol.

[103] Barta SK, Lee JY, Kaplan LD, Noy A, Sparano JA (2012). Pooled analysis of AIDS malignancy consortium trials evaluating rituximab plus CHOP or infusional EPOCH chemotherapy in HIV-associated non-Hodgkin lymphoma. Cancer.

[104] Barta SK, Xue X, Wang D (2013). Treatment factors affecting outcomes in HIV-associated non-Hodgkin lymphomas: a pooled analysis of 1546 patients. Blood.

[105] Dunleavy K, Little RF, Pittaluga S (2010). The role of tumor histogenesis, FDG-PET, and short-course EPOCH with dose-dense rituximab (SC-EPOCH-RR) in HIV-associated diffuse large B-cell lymphoma. Blood.

[106] Sparano JA, Lee JY, Kaplan LD (2010). Rituximab plus concurrent infusional EPOCH chemotherapy is highly effective in HIV-associated B-cell non-Hodgkin lymphoma. Blood.

[107] Sparano JA, Lee JY, Kaplan LD (2021). Response-adapted therapy with infusional EPOCH chemotherapy plus rituximab in HIV-associated, B-cell non-Hodgkin's lymphoma. Haematologica.

[108] Wilson MR, Bobillo S, Cwynarski K (2022). CNS prophylaxis in aggressive B-cell lymphoma. Hematology Am Soc Hematol Educ Program.

[109] Schmitz N, Zeynalova S, Nickelsen M (2016). CNS International prognostic index: a risk model for CNS relapse in patients with diffuse large B-cell lymphoma treated with R-CHOP. J Clin Oncol.

[110] Eyre TA, Djebbari F, Kirkwood AA, Collins GP (2020). Efficacy of central nervous system prophylaxis with stand-alone intrathecal chemotherapy in diffuse large B-cell lymphoma patients treated with anthracycline-based chemotherapy in the rituximab era: a systematic review. Haematologica.

[111] Ho G, Tan C, de Mel S (2021). Central nervous system (CNS) prophylaxis in antiCD20-CHOP treated DLBCL at intermediate to high risk for CNS relapse: a systematic review and meta-analysis. Crit Rev Oncol Hematol.

[112] Lin Z, Chen X, Liu L, Zeng H, Li Z, Xu B (2022). The role of central nervous system (CNS) prophylaxis in preventing DLBCL patients from CNS relapse: a network meta-analysis. Crit Rev Oncol Hematol.

[113] Tai WM, Chung J, Tang PL (2011). Central nervous system (CNS) relapse in diffuse large B cell lymphoma (DLBCL): pre- and post-rituximab. Ann Hematol.

[114] Ong SY, de Mel S, Grigoropoulos NF (2021). High-dose methotrexate is effective for prevention of isolated CNS relapse in diffuse large B cell lymphoma. Blood Cancer J.

[115] Nuvvula S, Dahiya S, Patel SA (2022). The novel therapeutic landscape for relapsed/refractory diffuse large B cell lymphoma. Clin Lymphoma Myeloma Leuk.

[116] Crump M, Kuruvilla J, Couban S (2014). Randomized comparison of gemcitabine, dexamethasone, and cisplatin versus dexamethasone, cytarabine, and cisplatin chemotherapy before autologous stem-cell transplantation for relapsed and refractory aggressive lymphomas: NCIC-CTG LY.12. J Clin Oncol.

[117] Neelapu SS, Locke FL, Bartlett NL (2017). Axicabtagene ciloleucel CAR T-cell therapy in refractory large B-cell lymphoma. N Engl J Med.

[118] Locke FL, Ghobadi A, Jacobson CA (2019). Long-term safety and activity of axicabtagene ciloleucel in refractory large B-cell lymphoma (ZUMA-1): a single-arm, multicentre, phase 1–2 trial. Lancet Oncol.

[119] Caron Jacobson M, Locke FL, Ghobadi A et al. 1187 long-term survival and gradual recovery of B cells in patients with refractory large B cell lymphoma treated with Axicabtagene Ciloleucel (Axi-Cel). In. 62nd ASH annual meeting and exposition 2020.

[120] Schuster SJ, Bishop MR, Tam CS (2019). Tisagenlecleucel in adult relapsed or refractory diffuse large B-cell lymphoma. N Engl J Med.

[121] Chong EA, Ruella M, Schuster SJ (2021). Lymphoma Program Investigators at the University of Pennsylvania. Five-Year outcomes for refractory B-cell lymphomas with CAR T-cell therapy. N Engl J Med.

[122] Schuster SJ, Tam CS, Borchmann P (2021). Long-term clinical outcomes of tisagenlecleucel in patients with relapsed or refractory aggressive B-cell lymphomas (JULIET): a multicentre, open-label, single-arm, phase 2 study. Lancet Oncol.

[123] Abramson JS, Palomba ML, Gordon LI (2020). Lisocabtagene maraleucel for patients with relapsed or refractory large B-cell lymphomas (TRANSCEND NHL 001): a multicentre seamless design study. Lancet.

[124] Locke FL, Miklos DB, Jacobson CA (2022). Axicabtagene ciloleucel as second-line therapy for large B-cell lymphoma. N Engl J Med.

[125] Kamdar M, Solomon SR, Arnason J et al. Lisocabtagene maraleucel versus standard of care with salvage chemotherapy followed by autologous stem cell transplantation as second-line treatment in patients with relapsed or refractory large B-cell lymphoma (TRANSFORM): results from an interim analysis of an open-label, randomised, phase 3 trial. Lancet. 2022 Jun 18; 399(10343): 2294-2308. doi: 10.1016/S0140-6736(22)00662-6. Erratum in: Lancet. 2022 Jul 16; 400(10347): 160. PMID: 35717989. 10.1016/S0140-6736(22)00662-6 35717989

[126] Sehn LH, Herrera AF, Flowers CR (2020). Polatuzumab vedotin in relapsed or refractory diffuse large B-cell lymphoma. J Clin Oncol.

[127] Sehn LH, Hertzberg M, Opat S (2022). Polatuzumab vedotin plus bendamustine and rituximab in relapsed/refractory DLBCL: survival update and new extension cohort data. Blood Adv.

[128] Salles G, Duell J, González Barca E (2020). Tafasitamab plus lenalidomide in relapsed or refractory diffuse large B-cell lymphoma (L-MIND): a multicentre, prospective, single-arm, phase 2 study. Lancet Oncol.

[129] Caimi PF, Ai W, Alderuccio JP (2021). Loncastuximab tesirine in relapsed or refractory diffuse large B-cell lymphoma (LOTIS-2): a multicentre, open-label, single-arm, phase 2 trial. Lancet Oncol.

[130] Kalakonda N, Maerevoet M, Cavallo F (2020). Selinexor in patients with relapsed or refractory diffuse large B-cell lymphoma (SADAL): a single-arm, multinational, multicentre, open-label, phase 2 trial. Lancet Haematol.

[131] Herrera AF, Chen L, Crombie JL (2022). Polatuzumab vedotin combined with R-ICE (PolaR-ICE) as second-line therapy in relapsed/refractory diffuse large B-cell lymphoma. Blood.

[132] Dickinson MJ, Carlo-Stella C, Morschhauser F (2022). Glofitamab for relapsed or refractory diffuse large B-cell lymphoma. N Engl J Med.

[133] Hutchings M, Mous R, Clausen MR (2021). Dose escalation of subcutaneous epcoritamab in patients with relapsed or refractory B-cell non-Hodgkin lymphoma: an open-label, phase 1/2 study. Lancet.

[134] Thieblemont C, Phillips T, Ghesquieres H (2022). Epcoritamab, a novel, subcutaneous CD3xCD20 bispecific T-cell-engaging antibody, in relapsed or refractory large B-cell lymphoma: dose expansion in a phase I/II trial. J Clin Oncol.

[135] Bannerji R, Arnason JE, Advani RH (2022). Odronextamab, a human CD20×CD3 bispecific antibody in patients with CD20-positive B-cell malignancies (ELM-1): results from the relapsed or refractory non-Hodgkin lymphoma cohort in a single-arm, multicentre, phase 1 trial. Lancet Haematol.

[136] FDA website. May 19, 2023. https://www.fda.gov/drugs/drug-approvals-and-databases/fda-grants-accelerated-approval-epcoritamab-bysp-relapsed-or-refractory-diffuse-large-b-cell. Accessed 26 June 2023.

[137] FDA website. June 15, 2023. https://www.fda.gov/drugs/drug-approvals-and-databases/fda-grants-accelerated-approval-glofitamab-gxbm-selected-relapsed-or-refractory-large-b-cell. Accessed 26 June 2023.

[138] Reddy A, Zhang J, Davis NS (2017). Genetic and functional drivers of diffuse large B cell lymphoma. Cell.

[139] Cutmore NH, Krupka JA, Hodson DJ (2023). Genetic profiling in diffuse large B-cell lymphoma: the promise and the challenge. Mod Pathol.

[140] Chapuy B, Stewart C, Dunford AJ, et al. Molecular subtypes of diffuse large B cell lymphoma are associated with distinct pathogenic mechanisms and outcomes. Nat Med. 2018 May; 24(5): 679–690. doi: 10.1038/s41591-018-0016-8. Epub 2018 Apr 30. Erratum in: Nat Med. 2018 Aug; 24(8): 1292. Erratum in: Nat Med. 2018 Aug; 24(8): 1290–1291. PMID: 29713087; PMCID: PMC6613387. 10.1038/s41591-018-0016-8 PMC6613387 29713087

[141] Schmitz R, Wright GW, Huang DW (2018). Genetics and pathogenesis of diffuse large B-cell lymphoma. N Engl J Med.

[142] Wright GW, Huang DW, Phelan JD (2020). A Probabilistic classification tool for genetic subtypes of diffuse large b cell lymphoma with therapeutic implications. Cancer Cell.

[143] Lacy SE, Barrans SL, Beer PA (2020). Targeted sequencing in DLBCL, molecular subtypes, and outcomes: a haematological malignancy research network report. Blood.

[144] Wilson WH, Wright GW, Huang DW (2021). Effect of ibrutinib with R-CHOP chemotherapy in genetic subtypes of DLBCL. Cancer Cell.

[145] de Leval L, Alizadeh AA, Bergsagel PL (2022). Genomic profiling for clinical decision making in lymphoid neoplasms. Blood.

[146] Lee JY, Kannan B, Lim BY (2022). The multi-dimensional biomarker landscape in cancer immunotherapy. Int J Mol Sci.

